# The Search for Origin of Young Adoptees—A Clinical Study

**DOI:** 10.3389/fpsyg.2021.624681

**Published:** 2021-07-23

**Authors:** Sara Skandrani, Marie-Rose Moro, Aurelie Harf

**Affiliations:** ^1^EA 4430, Université Paris Nanterre, Nanterre, France; ^2^Hôpital Cochin, Paris, France; ^3^Université Paris Descartes, Paris, France; ^4^Université Paris-Saclay, Saint Aubin, France

**Keywords:** adoptive family, adoptees, birth family, search for origin, social media, interpretative phenomenological analysis

## Abstract

In the current area of social media propagation, the adoptees' search for the birth family is increasingly reversed: more and more adopted adolescents are contacted directly by their birth parents, even if they did not search for them. This study explores the impact of these new forms of contact between adoptive family members and birth family members, through the qualitative analysis of clinical protocols of five adoptive families that sought counseling in a clinical setting devoted to international adoption. The interpretative phenomenological analysis revealed three themes. Two of them shared by the parents and their children: the feelings of anxiety and intrusion, as well as the feelings of guilt and debt. The last theme concerns only the parents: feelings of endangered family relations and can be divided into two sub-themes: feelings of threat by the birth family, feelings of an undermined parental role. Nevertheless, these new kinds of confrontations with the children's origins bear a potential of renegotiating adoptive family relationships and positive effects on mutual feeling of filiation. Exploring the impact of the search of adoptees by the birth family enables professionals involved in adoption to improve preventive and supportive work in the adoption process.

## Introduction

Young adoptees have been the subject of an increased interest in research in the last decades. A first research field focused on their mental health and risk of psychiatric illness. Many adoption studies have showed that adoptees are overrepresented in diagnosed people with a psychiatric disorder and in outpatient clinical settings (Hjern et al., 2002; Juffer and van Ijzendoorn, 2005; Wicks et al., 2010). A meta-analysis, conducted in 2016 (Behle and Pinquart, 2016) presented evidence for an increased risk of adoptees, compared to non-adoptees, for experiencing psychiatric disorders, contact with mental health services, or treatment in a psychiatric hospital.

These results were put in perspective by other authors (Miller et al., 2000; Harf et al., 2006). This overrepresentation of adoptees in outpatient clinical settings can be explained by the adoptive parents' propensity to more readily use mental health services, even in early stages of symptom-development.

Adoption-research is also interested in the impact of pre-adoption experiences on the children's social and emotional development (Verhulst et al., 1992; Rutter, 2005), but also on their family relations and the filiation process. Adverse pre-adoption experiences such as maltreatment by families of origin or neglect during institutional care in orphanages prior to adoptive placement, increase the risk of post-adoption psychosocial maladjustement, specially with externalizing problems (Gunnar, 2000; Wilson, 2003; Juffer and van Ijzendoorn, 2005; Tung et al., 2018). But post-adoption experiences, such as quality of parent–child relationship mediate these pre-adoption experiences to produce different outcomes (Howe and Fearnley, 2003; Skandrani et al., 2019). Post-adoptive family relationships represent therefore a protective factor on the adoptee's emotional and social development (van IJzendoorn et al., 2005; van IJzendoorn and Juffer, 2006). In this regard, an interesting field of research focuses on the parent-child relationship in adoptive family and the understanding of adoption.

### Increased Family Contacts in the Adoption Triad

Since the mid-90', adoptions have become increasingly open in the UK and the United States (Black et al., 2016; Farr et al., 2018), even if it concerns especially domestic adoptions. In this context, child adoption is understood as an extended kinship network, including, the adopted children, the adoptive parents and birth relatives (Reitz and Watson, 1992; Grotevant and McRoy, 1998).

Despite this trend in both countries, controversies surrounding birth family contact persist (Grotevant, 2012; Siegel, 2012). Research in this area highlights different benefits to increased openness in adoption (Berge et al., 2006). Contact between the adoptive family members and birth relatives is generally associated with positive outcomes in adjustment and relationships among this adoption triad (Grotevant et al., 2007, 2013; Siegel, 2012) and with satisfaction (Grotevant et al., 2007, 2013; Vandivere et al., 2009; Brodzinsky, 2011; Brodzinsky and Goldberg, 2016; Farr et al., 2018). Adoption-related communication promotes a positive identity development among adopted adolescents and emerging adults (Grotevant et al., 2007, 2013). Yet, contact and adoption communicative openness are not related to adoptees' externalizing behavior in adolescence or emerging adulthood (Grotevant et al., 2011).

In spite of these results in favor of an increased openness in adoption, uncertainty remains about how this should be achieved. The complexity of the process of renegotiating the boundaries of kinship following adoption for all those involved is not enough recognized (Jones and Hackett, 2012). A qualitative study with adolescent adoptees whose voices are usually less heard, suggest that some of them are satisfied without having contact with their birthmother (Berge et al., 2006). Although a majority of adopted adolescents desired more contact with their birthmothers, not all of them had this desire for more openness (Grotevant and McRoy, 1998; Berge et al., 2006).

However, these general results in favor of an increased contact between adoptive and birth families were mostly reported in the context of American and Britain domestic adoption. In contrast, the traditional model of adoption as a form of family substitution is still dominant in France, where closed adoptions are the norm (Skandrani et al., 2012). They imply a permanent and total break of the filliation's bond with the birth family. In comparison to the American adoption context, a second point is further noteworthy: In France, children are mainly adopted from abroad (421 adoptions in 2019^1^, primarily from Vietnam, Columbia, Thailand, Haiti, and Congo. These two observations highlight a different adoption context in France, which determines a different adoption practice, especially concerning the contact to the birth family, the adoptee's cultural belonging and their search for origins (Skandrani et al., 2012).

### Controversies Surrounding the Search of Origin

In France, the discussion about an increased openness in adoption takes place *via* controversies regarding the benefits and drawbacks of the search of origins in adoptive families. Some adoption professionals (Lévy-Soussan, 2002; Soulé and Lévy-Soussan, 2002) defined the search of origins as a never-ending search for affection and love. Those engaging in such a path are trying to compensate the loss that they have incurred through their adoption. Instead of enabling a process of grief and a positive identity development, the quest for the birth parents would obscure the real psychological challenge: the need of separation–individuation during adolescence. In contrast, other researchers support the understanding of the search of origin as a search of personal history (Delaisi de Parseval, 2002; Golse and Moro, 2017). The studies in this area revealed the importance of acknowledging the specificities of the adoptees' adoption story. Considering the birth and cultural context reflects one way to deal with the question of origin (Harf et al., 2015). Considering the birth family story and even the adoption-related losses can constitute an opportunity to integrate them in a coherent life narrative (Skandrani et al., 2019).

### Increased Contact Through Social Media

Contact is currently also becoming more common in international adoption with the enhanced use of internet and social media of birth and adoptive family members (Roby et al., 2005; Black et al., 2016). Adoptive families are increasingly confronted with the possibility of contact with birth family members, a contact sometimes initiated by the latter. Boundary challenges (Black et al., 2016) and concerns arise with these developments (Goldberg and Smith, 2011; Grotevant et al., 2013). Ambiguities, miscommunication (Hertlein, 2012; Black et al., 2016) and concerns about potential intrusion by birth family members into the adoptive private family lives (Neil, 2009; Goldberg and Smith, 2011; Black et al., 2016) can be consequences of social media contacts. This is even more a concern, when adoptive families are directly contacted by adoptees' birth relatives without having themselves reached out for them (Skandrani et al., 2020). This contact initiated by the birth family doesn't fall into the category of what could have been an open adoption. In the latter, the relation arrangements between the birth and adoptive families are determined in advance during the adoption process itself.

These new ways of contact in the adoption triad, i.e., contacts initiated by the birth relatives *via* internet and social media, remain mostly unstudied. In the situations at stake here, the contacts couldn't be anticipated and prepared by the adoption family members. Understanding their complexity is the goal of the present clinical study.

## Materials and Methods

The study explores the impact of new forms of contact between adoptees and their birth family, through the qualitative analysis of clinical protocols of five adoptive families.

### Data Collection

In a clinical setting devoted to international adoption (Harf et al., 2013), adoptive families searching for psychological counseling are received by two psychologists, a psychiatrist and a psychology trainee for a preliminary evaluation, before being referred to a specific therapeutic setting. This evaluation process consists of three sessions, occurring once a month. Each step of this evaluation process takes place with the whole family, i.e., with the adoptee, his/her adoptive parents and his/her siblings if he/she has some. In this clinical setting, the adoptees' ages range is between 3 and 20 years.

A series of specific topics are systematically raised as part of the evaluation process. First, the different family members are invited to narrate the adoption procedure as well as the first encounter between parents and children. They are questioned about their past and current contacts with the birth family members, about eventual pre-adoptive arrangements, about their relations and their personal experience in this context. They are also encouraged to expose their current concerns about the family relations, the adoptive children's psychological outcomes/adjustment and their understanding of the difficulties that are faced. Each session is transcribed *in vivo* by the psychology trainee, after obtaining the family's agreement.

The family's questioning around the child's origin, especially during adolescence becomes often apparent during these sessions, addressed by the children themselves or by their parents. This clinical experience showed an enhanced parental concern about contacts with the birth family through the social media and their questioning about boundaries (Skandrani et al., 2020).

To further study the implications of these new forms of contact with the birth family on the family dynamic and the adoptee's psychological development and adjustment, the transcripts of this evaluation process were analyzed as part of a research protocol.

### Participants

Five families seeking clinical counseling in adoption participated in this clinical study. In all five families the couples were married. Parents' ages ranged from 35 to 49 years at the time of their children's adoptions and at the time of the interview from 47 to 60 years at the time of the interview. Parents lived in urban areas of France. All of them were college-educated professionals.

Each family has adopted one child: three were from Tahiti, one from Ukraine, and one from Haiti. Three were girls and two were boys. At the time of their adoption, their ages ranged from 1 day to 5 years. At the time of the clinical interviews, they were aged 9–16 years (see Table 1).

**Table 1:** Participants' description (All names have been changed).

**Name**	**Age**	**Age at adoption**	**Country of origin**	**Type of contact**
Diana	16	1 month	Tahiti	The biological sister contacted the adoptee through social media
Pierre	15	1 day	Tahiti	The biological sister contacted the adoptee through social media
Marie	15	6 months	Tahiti	The biological sister contacted the adoptee through social media
Daria	14	4 years	Ukraine	The biological sister contacted the adoptee through social media
Paul	9	5 years	Haiti	The biological mother contacted the adoptive mother through social media

The reasons reported by the families for reaching out for adoption professionals were relationship difficulties in the adoptive family (*n* = 5 families), as well as adoptees' internalizing problems: anxiety (*n* = 5), sadness (*n* = 3), and suicide attempt (*n* = 2).

Each family came three times in clinical counseling. The sessions took place over a period of three months. After the evaluation process, all the families were referred to a specific clinical setting devoted to international adoption, for therapeutic support. Further, four of the adoptees (except the 9 years old Paul) were invited to seek individual psychotherapy.

Each session was transcribed during the clinical encounter. A total of 15 sessions were then analyzed.

### Data Analysis

These 15 clinical transcripts were analyzed by two independent researchers (who were different from the professionals involved in the clinic setting), according to the Interpretative Phenomenological Analysis (Smith and Osborn, 2008).

This qualitative research method allows the exploration of the adoptees' and adoptives parents' personal experiences and unique representations of the question of origin as well as of their contact with the birth family, through a detailed examination of their personal perceptions and lived experiences. Hence, an in-depth qualitative analysis was conducted. Through an iterative inductive process, the researchers proceeded to a detailed case-by-case study of each clinical transcript. They began with several close, detailed readings of each clinical transcript to gain a holistic perspective, noting points of interest and significance. Through a step-by-step analysis, analytic themes emerged, which were described, as well as their interconnections, while keeping a link back with the original clinical transcripts. This process produced a coherent and ordered table of emerging themes. This data analysis procedure was inductive, since the analysis of the international literature on this specific subject was performed in the aftermath.

The sample size was determined by meaning saturation: we stoped including new families, when no additional information emerged from the data (Wilson, 2015; Hennink et al., 2017). We used this type of data saturation, as we aimed for an in-depth understanding of the phenomenon under investigation—i.e., the search of origin, when initiated by the birth family—from the perspective and experiences of adoptees and adoptive parents. Clinical transcripts ensure to gather deep, rich, detailed, and relevant data. Thus, the meaning saturation was reached, when no further insights were originating from our data.

### Validity

The researchers involved in this study are specialized in international adoption, family therapy, and trauma. The researchers codings were compared in order to insure the validity of this research.

Two trained researchers (SS and AH) independently coded and interpreted the clinical transcripts. The emerging codes were repeatedly discussed with another research team member (MRM) who had read the transcripts. These discussions allowed to identify additional themes in the data that might not yet have been described in the codes. It enabled the researchers to complete or modify the coding in order to increase the consistency and coherence of the analysis. It was thus ensured that the themes were accurately identified and reflected the data. Through this process, systematic differences, due to variations in interpretation, were eliminated. Validity was also enhanced by clearly distinguishing between the patients' discourse and the researchers' interpretation (Smith and Osborn, 2008).

### Ethical Statements

A clinical study such as this raises many ethical issues for consideration. Ethical approval was given by the Comité d'Evaluation de l'Ethique des projets de Recherche Biomédicale (CEERB) du Groupe Hospitalo—Universitaire Nord, on the 29th of March, 2011 (Institutional Review Board N° IRB00006477).

Parents and adoptees were fully informed about the voluntary nature and the goals of the study. Written informed consent was obtained from all participants included in the study before recording the sessions und using the transcripts. Participants were informed that all responses would be confidential, that the transcripts would have no identifying information, and that they would be free to withdraw at any time, without any incidence on the process of clinical counseling they were involved in. All identifying informations were removed from the transcripts, and participants' anonymity further ensured through disguising or withholding descriptive data.

## Results

Four adoptees in our clinical study were contacted by their biological siblings *via* social media. In one family only, the mother was the one contacted directly by the birth mother. These contacts took place either in French (Haiti and Tahiti) or in English (Ukraine).

Three themes emerged from the interpretative phenomenological analysis of these 15 clinical protocols: overwhelming feelings of anxiety and intrusion, feelings of guilt and debt, parental feelings of threat and insecurity (see Table 2). These themes will be illustrated by patients' quotations.

**Table 2:** Themes and sub-themes.

**Themes and sub-themes extracted from the analysis**	**Adoptees**	**Adoptive parents**
Overwhelming feelings of anxiety and intrusion	5	10
Feelings of guilt and debt	5	10
Feelings of endangered family relations	–	10
Parental feelings of threat by the birth family		10
Feelings of an undermined parental role		10

### Overwhelming Feelings of Anxiety and Intrusion

The first emerging theme concerns the feelings of anxiety and intrusion experienced by the adoptees after their birth family and especially their birth siblings reached out for them.

The adoptees reported being overwhelmed by the suddenness of this contact with their birth family, especially as they did not seek for them.

“Before, I didn't think of meeting them or even looking for them. I am here, I mean, my life is here” (Marie, 15).

“She [the older biological sister] contacted me… Just like that. And she told me my story. I mean, I knew the story, my parents told me, but she told me other stuff… And then my mother, my birth mother called me. It was…” (Diana, 16).

Diana can't continue her story, she is overwhelmed by her emotions. Even 2 years after this first contact and its persistence since, she can't talk about it without this overflow of emotions. Especially as she wasn't prepared for this virtual encounter. She was not, or not yet, thinking about her birth family.

“I mean, I was not thinking about them (the birth family). Sometimes we talked about going there, with my parents, some day. Sometimes I wanted to go to Tahiti and sometimes not. But I was not thinking about them.”

Diana's mother remembers that her daughter was “dazed” afterward and “chocked.” And she didn't know what to do.

“But I think afterwards it became difficult with Diana, she started fighting with us. And I think it's related.” Diana nods to her mother's description.

Pierre (15) reported a suicide attempt close after the first contact with his biological sister from Tahiti.

“I tried to kill myself, because my girlfriend broke up with me… Especially since my sister contacted me *via* facebook. She just said that she is my sister. I learned then that our father, i mean my biological father, just died too.”

Pierre was completely unprepared for this contact and even more for the announcement of his biological father's death. In this context, the breakup with his girlfriend was a trigger for the expression of his overwhelming anxiety.

Daria was adopted in Ukraine when she was 18 months old, after her mother died. When she was 13, she was contacted by an elder biological sister *via* facebook. Her parents and her brother reported her being afterward “outside of reality.” She started going to an elder neighbor, spending all her time there and telling her lies about her parents' behaviors toward her. She alarmed her neighbor so much that the woman reported Darias parents to the social service for abusing Daria. A subsequent social investigation shed light on Daria's lies about her parents' behaviors. Thereafter, the whole family agrees that Daria was very disturbed by the contact with her biological sister.

“She [the older sister] broke into my life. After that, everything went upside down.”

All five families reported not having been prepared for these contacts. The adoptive parents report having always been open to discuss with their children their pre-adoptive history and to share with them what they knew about the birth parents. But they had no active contact with any member of the birth family since the adoption.

“We have a flexible brain and a flexible heart” (Pierre's mother).

But when the link to the birth family was created, without them—neither the parents nor the children—seeking for these contacts, they experienced the latter as intrusive and even invasive.

“It's too much. I mean, she [the elder biological sister] is always coming virtually in our family. That does not help Daria to make plans here, to concentrate on her future school choices. How to stop her?” (Daria's father).

### Feelings of Guilt and Debt

The analysis of the clinical protocols revealed feelings of guilt and debt, experienced by both the adoptees and their parents.

After acknowledging his birth father's death, Pierre expressed a feeling of guilt toward him. In his adoption story, this man was held responsible for his abandonment and adoption. That's why Pierre was always angry with him, until he brutally discovered his death.

“I mean, when I found out, I couldn't go and piss on his grave.”

Suddenly feelings of debt toward this man who gave him life, are experienced by Pierre and led to much anxiety.

Daria is very concerned with the life of her biological sister: she is pregnant although still very young, she is beaten by her boyfriend and living in very precarious life conditions. The 14 years old Daria tries to give her advice regarding her pregnancy and her life, is always very anxious when she hears about the couple's fighting. She even tries to contact the boyfriend to tell him to stop harming her biological sister.

“For me, life is simple. But for her, everything is difficult. I want to help her, I want to be there for her.”

She is experiencing feelings of guilt for her “better” life conditions, even if she is herself in psychological distress—she is very anxious, sometimes even hurts herself. Her sister's alarming situation is too heavy to bear for a 14 year old girl.

Paul's mother was contacted by her son's biological mother *via* facebook. She is very worried that this woman could ask her for money, although the biological mother didn't say anything in this regard.

“I don't want to begin such a relationship. But I can understand. I mean, she doesn't have anything, she must be so poor, but we aren't.”

Through her worries, she is expressing her ambivalent feelings regarding what she owes to this birth mother. This contact situation that her family didn't seek reveals her feelings of debt toward her.

After the contact with her birth family has been set through her biological sister, Diana now wants to travel to Tahiti. She is working to pay for her airplane ticket. To her parents, who are trying to postpone the trip, she answers:

“It's urgent. I can't prepare for the trip, you can't prepare yourself for such an encounter. It will be associated with a lot of emotions, it's logic, you can't avoid it.”

Encountering her birth family is “urgent” because she owes them a lot and this contact has reminded her of that. That's why it is so important for her to pay for the trip by herself and also to go there by her own.

Now that she has a regular though virtual contact with her biological sister, Marie wants to go and visit her in Tahiti. Even if she will only be graduating in at least 4 years, she is already planning to move afterwards to Tahiti for a few months to help the people there through a humanitarian internship.

“I want to give a little bit of what I have. And to my sister too.”

What does she want to give to her sister? Why does she want to give her something? Through this imprecise sentence “And to my sister too,” she is expressing indirectly her feelings of guilt and debt toward her.

### Feelings of Endangered Family Relations

The third emerging theme concerns only the parents. They experience and perceive the contacts initiated by birth family members as endangering their relations to their children. Their narratives reveal a difficult coexistence for them, of both filiations—the adoptive and biological one. The two kinds of relationships are experienced as competitive. This theme can be divided into two sub-themes: Parental feelings of threat by the birth family and feelings of an undermined parental role.

#### Parental Feelings of Threat by the Birth Family

The birth family, breaking in the family life without warning nor preparation or desire to let them in, was experienced by the parents in our clinical sample as a threat.

Paul's mother felt endangered by potential demands of the biological mother, even if the latter didn't make them: requests for money but also for help to come to France for example.

When Pierre's birth mother contacts him to criticize his misbehavior at school and with his peers, his mother intervenes:

“She should mind her own business! If she gives him [Pierre] away, she gives him away, it's no longer her business.”

Even if she agreed to this contact, initiated by the birth family, she experiences it in the long run as intrusive and threatening. She needs to warn her son:

“Nobody is waiting for you at the other end of the world. Everything is taking place here.”

Diana's father is fearing the negative effects of the relation with the biological sister on his daughter. Even if Diana denies it, he thinks that she is disturbed by this contact. He even makes it responsible for the psychological distress his daughter is experiencing since several weeks.

Daria's parents have a similar opinion: all the family's dysfunction began after the relation with her biological sister started. This passively experienced contact had a shattering effect on their family. The parents felt they almost lost their daughter, after a social investigation took place, as Daria pretended being abused by her parents.

#### Feelings of an Undermined Parental Role

The feelings of endangered family relations are linked to a second sub-theme, which also concerns the parents only: their loss of confidence in their parental function and, in this context, their feeling of insecurity.

Paul's parents fear not being able to protect their son from all the harm he could experience in the outside world. This anxiety is related to the biological mother's search for them.

“It's difficult to accept that we aren't able to protect Paul from all the dangers in the outside world. He is rejected by his peers, even his teachers are not very nice to him. And when his biological mother contacted us, we were not able to avoid this neither.”

Pierres' father expresses a similar feeling: he failed in his parental role—screening the informations given to his son by the birth family.

“The brutal announcement of the death of his biological father, we were not able to avoid that. But we should have been.”

Faced with this parental insecurity, some adoptees try to reassure them.

In Diana's opinion, her parents are opposed to her trip to Tahiti because they feel insecure concerning their relation to her. They would worry that their bond will be undermined by the bond between her birth family and herself.

“My parents fear losing me. They fear that I won't come back. That's why my mother wants to come with me. But I want to go alone. As my birth mother searched for me, my mother thinks she wants to keep me now.”

She tries to reassure them concerning the strength of their relation.

“I didn't grow up with my birth family. They don't know me, they weren't there for me. It's completely different from my adoptive family.”

Marie's parents regret that they weren't capable from preventing a contact with the birth family. Otherwise, they think, their daughter wouldn't have wanted to go to Tahiti. In this context, Marie tries to convince them about their essential role in her life.

“Yeah, I didn't want to go to Tahiti, before my sister reached out to me. But now I want to go, but I will come back. My life is here, with them [the adoptive parents]. [She turns around smiling at them] Don't worry!. ”

Through their narratives the parents are expressing their anger toward the birth family members as well as their fear of losing their children and their bond with them, as if both relations—to the adoptive family and to the birth family—couldn't coexist.

## Discussion

The interpretative phenomenological analysis revealed three themes. Two of them shared by the parents and their children: the feelings of anxiety and intrusion, as well as the feelings of guilt and debt. The last theme—feeling of endangered family relations—is experienced by the parents only.

### The Emergence of Birth Family

In our clinical experience, the contacts *via* social media initiated by the birth family take place more and more often. In these situations, the quest of origins is reversed, as it is not started by the adoptive family or the adoptees themselves but by the birth family. In the adoptive family, this initiative can be experienced as intrusive and even invasive, since it was not prepared. The adoptees are alone, in front of their computer or phone when the message suddenly pops up. This lack of preparation has therefore an important impact on the way the search of origin can be deployed.

In all adoptive families, the question of origin becomes at some point an issue for the adoptees themselves and/or for their adoptive parents (Skandrani et al., 2020). Many studies (Grotevant et al., 2007, 2013; Hawkins et al., 2007; Le Mare and Audet, 2011; Siegel, 2012) show the positive effect of an open discussion or at least the possibility to address this subject in the family. Some adoptees will ask questions directly concerning their birth mother or birth family, others will address them indirectly through an interest in the culture of origin (Harf et al., 2015; Benoit et al., 2018). Other adoptees will take a trip to the country of origin, visit the orphanage or the location of their abandonment; or just plan the trip and always postpone it (Mazeaud et al., 2019). The quest of origin can thus only be fantasized, without never taking place through direct action.

These different ways of approaching and integrating the question of origin in the adoptees' identity construction are impaired when a contact is initiated by the birth family and is taking place through social media. The adoptees have no longer the possibility to confront this question of origins at their own pace, in discussion with their adoptive parents but also with others, like siblings, peers or adoption professionals.

Moreover, adoptees cannot prepare themselves for the emotional overflow they might experience or protect themselves from it. They are put in a passive, even helpless, position, a position that bears the risk of a shattering and dazing effect on them. This sudden emergence of birth family members through social media brings up the issue of its traumatogenic effect on unprepared adoptees (Skandrani et al., 2020). This applies even more in a context in which openness in adoption is rare if not non-existent, such as in the French context. Cultural differences in the representation and process of adoption between different countries are reported in several studies (Rushton and Minnis, 1997; Skandrani et al., 2012; Harf et al., 2015). Contemporary child adoption in the UK and USA has been conceptualized as an extended kinship network of adopted children, birth relatives and adoptive parents (Reitz and Watson, 1992; Grotevant and McRoy, 1998). This contrasts sharply with the French model of adoption as a form of family substitution. The absence of open adoption in the French context and the subsequent lack of approaches to think and deal with relations with the birth family deprives the adoptees and their parents of possibilities to make sense of these new experiences.

In our clinical study the adoptive parents worry about birth parents interfering in their parental role. They express feelings of anxiety, insecurity, and even threat. They worry that these contacts confuse their child, undermining his/her emotional well-being but also their protective role as parents. They feel neutralized in their parental function of introducing the world to their child in small doses, according to his/her needs and possibilities (Winnicott, 1957), of screening the child's relation to the outside world, of protecting them if necessary. The adoptive parents cannot support their children and walk them through their, often necessary, questioning concerning their origins. Their function as supportive and protective environment for their children is impeded (Winnicott, 1957). As our clinical study showed, they lose confidence in their parental abilities and experiences and become insecure in their relation to their children. The intensity of these feelings of helplessness seems specific to these contact circumstances, as parents don't express the same level of intensity in other situations, neither in real life nor on social media. Sometimes the adoptees must reassure them regarding the continuity and stability of their relationship. It seems that it is not only the contact with the birth family that is responsible for this insecurity but also the way it took place and its meaning for them. Our clinical results showed how this passive, even helpless position can trigger feelings of intrusion and threat by the birth family. These contacts initiated by the birth family are experienced by the parents as a danger for their family relations, as both filiations seem unable to co-exist for them. As stated above, this is probably even stronger in the French context in which the adoption model of family substitution is dominant, depriving adoptive parents of relationship models between the birth family and the adoptive family (Skandrani et al., 2012; Harf et al., 2015).

The new kind of relation to the birth family exposed here has the potential of disrupting the relations in the family and the roles of the children and their adoptive parents. Moreover, the virtual aspect of this beginning relation, taking place exclusively or for a long time through social media only, deprives everyone of the supportive, warm, real aspect of relations. The immediacy of the contact and the communication bears an invasive and *overwhelming* aspect, making the integration of this part of their life narrative particularly challenging for the adoptees.

### Re-negotiating the Adoptive Family Relationships

Our results show some negative effects of these contacts initiated by the birth family, such as feelings of anxiety and intrusion and consequent behavioral issues, confirming parental concerns expressed in other studies (Turkington and Taylor, 2009).

However, our results reveal also how the feelings of debt and even guilt triggered by these contacts initiated by the birth family, allow some adoptees to renegotiate relationships within their adoptive family. After experiencing initial overwhelming feelings of anxiety and intrusion, adoptees often take an active part in shaping the relation to their birth family members: they engage in a more or less frequent exchange with them through social media (Daria, Pierre, Marie, and Diana), plan to go visit them (Diana, Marie), make space for them in their daily life (Daria, Pierre).

At the same time, they reaffirm their feelings of belonging to their adoptive family. When confronted with their parents' feelings of insecurity, they try to reassure them about their filiation and bond to the adoptive family: Diana and Marie express their attachment to their “true” adoptive parents, even if they want to meet their biological family.

The parents themselves, even if challenged in their parental role, claim their child's belonging to their family and the primacy of their current family over the birth family. They insist on and make explicit their attachment to their child, which is even more important in the context of family distress and conflicts, they may often experience. They assert being the parents of these children. By doing so, they offer already an emotional and appeasing support to their children, as they anchor them in the here and now of their adoptive family. This result highlights, that the way in which adopted children and parents react to contact's require from the birth relatives, as well as whether they are able to integrate the past and the present in a coherent history, depend on the relationship they have built together since the beginning of adoption.

Therefore, the contact by the birth family can be in the long run an opportunity to reaffirm the mutual attachement (Pace et al., 2015, 2019) between children and parents in adoptive families and to redefine the family boundaries. A study exploring adolescents' feelings about openness in adoption revealed the difference between relationships to birthmothers and to adoptive parents: for many adolescents, the relationships to the birthmothers were more like friendships and did not replace their relation to adoptive parents (Berge et al., 2006).

The contact with birth families can represent an opening for parents and children to speak, sometimes for the first time, sometimes again, about their family construction, their relationships, and feelings of belonging. It supports an openness in communication about the adoption procedure, whose positive effects on the child's psychological outcomes are largely reported in the research (Grotevant et al., 2007, 2013; Hawkins et al., 2007; Le Mare and Audet, 2011; Siegel, 2012). These contacts with birth family members and the new informations and insights they potentially bear can sustain adoptees in the elaboration of their life narrative (Delaisi de Parseval, 2002; Golse and Moro, 2017).

Our results show that these new challenges faced by adoptive families confirm the dynamic aspect of adoptive relationships and the necessity of an ongoing process of active negotiation and involvement of those affected by adoption (Jones and Hackett, 2012).

### The Issue of Boundaries

The matter of openness in adoption applies differently when the contact is initiated by the birth family itself. As stated earlier, the adoptees cannot think about the matter by themselves and with the support of their adoptive parents. They are confronted with the question of origins without having raised it themselves.

In this context, the issue of boundaries seems a central one. The notion of “boundaries” has always been important in adoption, where it refers to limitations of engagement of adoptive and birth family members (Grotevant et al., 2013). Even when the adoptive parents are in favor of openness in adoption, they face challenges in navigating between boundaries and contacts (Goldberg and Smith, 2011).

The feelings of anxiety, insecurity and even threat expressed by the parents in our clinical sample echo the findings of other studies exploring open adoption (Turkington and Taylor, 2009). Black et al. (2016) reported parental boundary concerns related to contact *via* technology, and more specifically concerns about potential intrusion by birth family members into their own private family lives.

The use of internet and social media creates new challenges in defining boundaries in adoptive family relationships (Hertlein and Blumer, 2014). The present results underline the need for specific support for the adoptive parents and adoptees, making them aware of the possibility of contacts by the birth family, preparing them for the renegotiation of boundaries, and address more generally the matter of openness in adoption.

The analyses presented here are however limited by the clinical aspect of our sample. This subject should be explored in a general population survey, using mixed—i.e., qualitative and quantitative—methods. Such a study design would allow to audio record and then transcribe the participant's narrative and not only to transcribe it *in vivo* during the clinical session. A second limitation, which is frequent in qualitative research, concerns the difference between families who volunteered in this study and those who declined. The participants in our study may have more psychological resources than other families in facing the challenges borne in contacts initiated by birth family members. Furthermore, future studies could include the third part of the adoption triad: the birth family. Their reactions to the new possibilities created by social media, as well as their related emotions, could be explored.

## Conclusion

The results of this clinical study show the complexity of the contacts to birth family members, when they are not initiated by the adoptees themselves or their parents. Although they trigger feelings of anxiety, intrusion and guilt in both, adoptees and their parents as well as feelings of threat and insecurity specifically in adoptive parents, they bear the potential of renegotiating adoptive family relationships and positive effects on mutual feelings of filiation. The second major issue highlighted by our results, concerns the role of social media in the first contact between adoptees and birth family members. This mean of communication carries the risk of a non-prepared, invasive, and exclusively virtual aspect of the relation to the birth family.

Exploring this subject enables professionals involved in adoption to improve the conditions of these contacts, which present the risk of being experienced in a passive, if not, helpless position. Adoption professionals can prepare adoptees and their parents to the increasing possibility of these contacts through social media and to the related challenges in terms of child well-being, parent-child relation and family boundaries. They can also support adoptees and their parents in their search for appropriate online relationships with the birth family members.

This is even more important since it is not adoptees with poor relationships with adoptive parents that are most satisfied with birth family contact (Farr et al., 2014). At the same time, openness is not desired by all adoptees and some are happy with their lives without it (Berge et al., 2006). The diversity among the needs and desires of adoptive families' members support our conclusion that it isn't the contact with birth families that is at stake, but the way it takes place.

## Data Availability Statement

The raw data supporting the conclusions of this article will be made available by the authors, without undue reservation.

## Ethics Statement

The studies involving human participants were reviewed and approved by Comité d'Evaluation de l'Ethique des projets de Recherche Biomédicale (CEERB) du Groupe Hospitalo—Universitaire Nord, on the 29th of March 2011 (Institutional Review Board N° IRB00006477). Written informed consent to participate in this study was provided by the participants' legal guardian/next of kin.

## Author Contributions

Two trained researchers SS and AH, independently coded and interpreted the clinical transcripts. The emerging codes were repeatedly discussed with another research team member M-RM who had read the transcripts. All authors contributed to the article and approved the submitted version.

## Conflict of Interest

The authors declare that the research was conducted in the absence of any commercial or financial relationships that could be construed as a potential conflict of interest.

## Publisher's Note

All claims expressed in this article are solely those of the authors and do not necessarily represent those of their affiliated organizations, or those of the publisher, the editors and the reviewers. Any product that may be evaluated in this article, or claim that may be made by its manufacturer, is not guaranteed or endorsed by the publisher.

## Footnotes

^1^Government source: https://www.data.gouv.fr/fr/datasets/adoptions-internationales-pays-dorigine-zone-geographique-tranche-dages-et-procedures/
